# Using near infrared measurements to evaluate NaCl and KCl in water

**DOI:** 10.1177/0967033518821834

**Published:** 2019-01-07

**Authors:** Reisha D Peters, Scott D Noble

**Affiliations:** College of Engineering, University of Saskatchewan, Saskatoon, SK, Canada

**Keywords:** NaCl, KCl, salinity, spectroscopy, water, absorbance

## Abstract

Spectral differences between aqueous solutions of NaCl and KCl have received minimal attention in previous research due to strong similarities between the two salts and the lack of motivation to differentiate between them. Correlations between salinity and absorbance have been developed previously with varying degrees of linearity but have not been tested to saturation. This work will demonstrate that correlating spectral measurements and the concentration of NaCl and KCl in water can be extended up to the saturation point of both salts and that solutions of these salts with unknown concentrations can be distinguished. Spectral data for samples of NaCl and KCl in single-salt solutions were collected up to saturation and correlations were developed for differentiating between solutions of the two species. These correlations were able to correctly identify the solution type for all solutions in the test set and estimate their concentrations with an average error of 0.9%.

## Introduction

Stretching and bending vibrations between water hydrogen and oxygen cause absorption in mid infrared range of the light spectrum. These absorption bands have overtones which extend into the near infrared and visible range.^1^ The shape and position of these overtones are affected by temperature, pressure, salinity, and other impurities. These factors also affect the physical properties of the water including hydrogen bonding. The addition of salt to water has a number of effects on the absorption spectrum. The most obvious change would be the addition of the absorption exhibited by the individual ions. While NaCl and KCl do not absorb strongly in the visible range, there are some absorption features in the ultraviolet range that add to the water absorption.^2,3^ Fluorescence in the visible range is present with ultraviolet excitation but is very minimal for NaCl and KCl.^2^ There are no ion absorption features for these salts in the near- or short-wave infrared regions.^4^

As NaCl and KCl are added to water, the concentration of water decreases. This overall decrease in water molecules in a given volume causes the absorption bands to be reduced and, subsequently, the absorption harmonics present in the near infrared and visible range to be reduced. This explains why transmission of light through a salt water sample increases in certain wavebands as the salt concentration increases. NaCl and KCl cause changes in the structured arrangement of water molecules.^5^ These salts cause a shift in makeup of hydrogen bonding toward higher absorption, longer wavelength bands in the stretching vibrational absorption bands as more structure results in stronger, better aligned hydrogen bonds.^6,7^ The different interactions between the NaCl and KCl molecules with water may cause species-specific shifts in absorption peaks, given the differences in size and electronegativity of the Na^+^ and K^+^ ions.

Hirschfeld^4^ investigated the effect of ion concentration on the water absorption bands in the near infrared region finding a linear correlation. The concentrations under investigation were all less than 0.651 mol/L (10% of the maximum) and extrapolation of these findings would be inappropriate. Röttgers et al.^8^ considered salinity to have a linear effect on absorption at all wavelengths in the visible and near infrared (NIR) regions but were only concerned with concentrations up to that of seawater. Lin et al.^9^ also used NIR spectroscopy to investigate various electrolytes in aqueous solutions. The focus of their research was identifying the species present in the water for samples containing one, two, or three electrolytes. That study only considered a single concentration of 0.5 mol/L for each solution and included many species other than NaCl and KCl, but it provides confirmation that different electrolytes provide qualitatively different changes to the water absorbance spectrum. It is important to note that Lin et al. did not consider differences in spectral shape between NaCl and KCl and that 0.5 mol/L KCl produces a greater change in the spectrum than 0.5 mol/L NaCl. The specific differences of these two salts at various concentrations was not explored.^9^

The spectra of NaCl and KCl have been described as being very similar in previous studies.^10,11^ This is due in part to their similar number of water molecules per solvated molecule.^12^ Some works have even classified KCl, along with other salts, into a subcategory of NaCl due to their similarities.^13^ Studies have considered the effects of high concentration of NaCl and KCl in the IR region of the spectrum, but the changes in the NIR were not reported (specifically 800–1400 nm as is the interest of this work).^11,13^

Though the use of spectroscopy for analysis of water and salt water samples is not a novel concept, the research on the subject has been largely limited to the concentration range typical of sea water.^8^ Due to the similarities between NaCl and KCl, they have often been assumed to have little to no differences in their effects on the absorbance spectrum of water. In the water absorption overtone region, correlations between salinity and absorbance have been developed up to about 10% of saturation using the water absorption overtones but these results have not been extended to the high concentration range and have not focused on species differentiation.

The results of this current research demonstrate that correlations between absorption and concentration of NaCl and KCl in water can be extended up to the saturation point of both salts and that solutions of unknown concentrations of either salt can be distinguished from each other based on NIR absorbance spectra. Spectra of NaCl and KCl samples up to saturation are presented to show the effects of each salt on the absorbance. Using the data obtained from these measurements, correlations were developed that can accurately classify whether a sample contains NaCl or KCl and determine the concentration of that salt in the solution. Specific wavelengths were chosen for these correlations based on relative sensitivities between absorption changes and species-specific concentrations. A simple approach for species determination was desired so linear correlations and a few select wavelengths were used. The results of this work will provide a foundation for future development of correlations that can determine concentrations of salts in multi-salt solutions at high concentrations.

## Experiment design

Solutions of NaCl and KCl with concentrations up to near-saturated values were required to characterize the effects of each salt as they approached their limits of solubility. Once each salt was characterized individually, the differences in absorbance changes were used to differentiate between solutions of the two salts.

### Mixing of solutions

Solutions of NaCl and KCl were prepared using reagent-grade NaCl (99.9%) and KCL (99.6%) from Fisher Scientific (Ottawa, ON, Canada) and deionized water. Solutions were mixed in Class A volumetric flasks and both salt and water masses in the mixture were recorded. This allowed for measurement of both molar and the g/g_water_ concentrations of the samples. In much of the literature, solubility of NaCl and KCl in water is given in grams of salt per 100 grams of water. Spectral absorbance, however, is based on moles per unit volume. Conversion between these two solubility measurements was not performed as the concentration of some solutions is high. Both measurements are therefore provided here. Tables 1 and 2 describe the quantities of salt and water in each sample. The two lowest concentration samples for each salt species were made using 100 mL flasks as the quantities of salt were much smaller than the higher concentration samples. For reference, at 25℃, saturation is 0.357 g/g_water_^−1^ for NaCl and 0.358 g/g_water_ for KCl.^14^

**Table 1.** Solution details for NaCl (molar mass NaCl: 58.44 g/mol).

Water (g) ±0.001 g	NaCl (g) ±0.001 g	Volume of solution (mL)	g NaCl/100 g water	Concentration (mol/L)
95.585	8.676	98.70 ± 0.08	9.077 ± 0.001	1.504 ± 0.001
92.795	15.95	98.70 ± 0.08	17.188 ± 0.001	2.765 ± 0.002
45.566	11.802	50.00 ± 0.05	25.901 ± 0.002	4.039 ± 0.004
44.912	13.181	49.90 ± 0.05	29.349 ± 0.002	4.520 ± 0.005
44.44	14.335	49.90 ± 0.05	32.257 ± 0.002	4.916 ± 0.005
44.191	14.859	49.90 ± 0.05	33.624 ± 0.002	5.095 ± 0.005
44.113	15.094	49.90 ± 0.05	34.217 ± 0.002	5.176 ± 0.005
43.985	15.416	49.90 ± 0.05	35.048 ± 0.002	5.286 ± 0.005

**Table 2.** Solution details for KCl (molar mass KCl: 74.55 g/mol).

Water (g) ±0.001 g	KCl (g) ±0.001 g	Volume of solution (mL)	g KCl/100 g water	Concentration (mol/L)
95.214	8.599	98.70 ± 0.08	9.031 ± 0.001	1.169 ± 0.001
92.288	15.921	98.70 ± 0.08	17.251 ± 0.001	2.164 ± 0.002
44.855	11.835	49.90 ± 0.05	26.385 ± 0.002	3.181 ± 0.003
44.168	13.279	49.90 ± 0.05	30.065 ± 0.002	3.570 ± 0.004
43.825	14.084	49.90 ± 0.05	32.137 ± 0.002	3.786 ± 0.004
43.689	14.416	49.90 ± 0.05	32.997 ± 0.002	3.875 ± 0.004
43.383	14.838	49.90 ± 0.05	34.202 ± 0.002	3.989 ± 0.004
42.867	15.308	49.90 ± 0.05	35.710 ± 0.002	4.115 ± 0.004

### Spectral absorbance measurements

All spectra were collected using a dual-beam spectrophotometer (Cary-5000, Agilent Technologies, Mississauga, Canada). Quartz cuvettes with a 10 mm path length were used for all measurements. No lids were used to cover the cuvette as temperatures were low (25℃) and scan times were short (less than 15 min). Evaporation from the cuvettes at this temperature and time span was undetectable at ±0.05 mg resolution. Solution temperature was moderated by a dual-cell temperature controller for both the sample and reference cuvettes. Samples were stirred during scans to help mitigate any crystallization on the cuvette wall for very high concentration samples. Spectra were collected between 180 and 1800 nm at 1-nm intervals with a 2-nm bandwidth. A target signal to noise ratio of 5000 was used with a timeout of 0.5 s. For each solution, scans were taken in triplicate with sample replacement between scans. The cell temperature accessory was set to 25℃ for all scans. Samples were given 5 min to reach an equilibrium temperature before the spectra were collected. Temperature monitoring for pure water samples indicated that this was ample time for a consistent and uniform temperature in the cuvette.

A zero/baseline correction was applied to all scans using two matched empty cuvettes (i.e., air-filled) for 100% transmittance and an empty cuvette in the reference beam path with the sample beam blocked for 0% transmittance. All spectra were measured with an empty cuvette as reference. A scan of deionized water at 25℃ was taken for all scanning sessions as a water reference. Water was not used in the reference cuvette because the concentration of water in the reference and the sample was not the same. Using an empty cuvette as the reference allowed for any absorbance from the cuvette walls to be accounted for but provided a more flexible set of data that could be compared to the water scan taken with the sample in later processing if desired.^12^

## Experimental results and analysis

Consistent with previous research, NaCl and KCl were observed to have very similar absorbance spectra. However, a few regions where the shape and intensity differ were observed. Absorbance data were collected from 180 to 1800 nm and showed good agreement with previous research in the ultraviolet range,^15^ visible,^16^ and near infrared.^5,7^ The most notable differences between NaCl and KCl were observed between 800 and 1400 nm, therefore, the subsequent portions of this work focus exclusively on this region.

**Figure 1.**
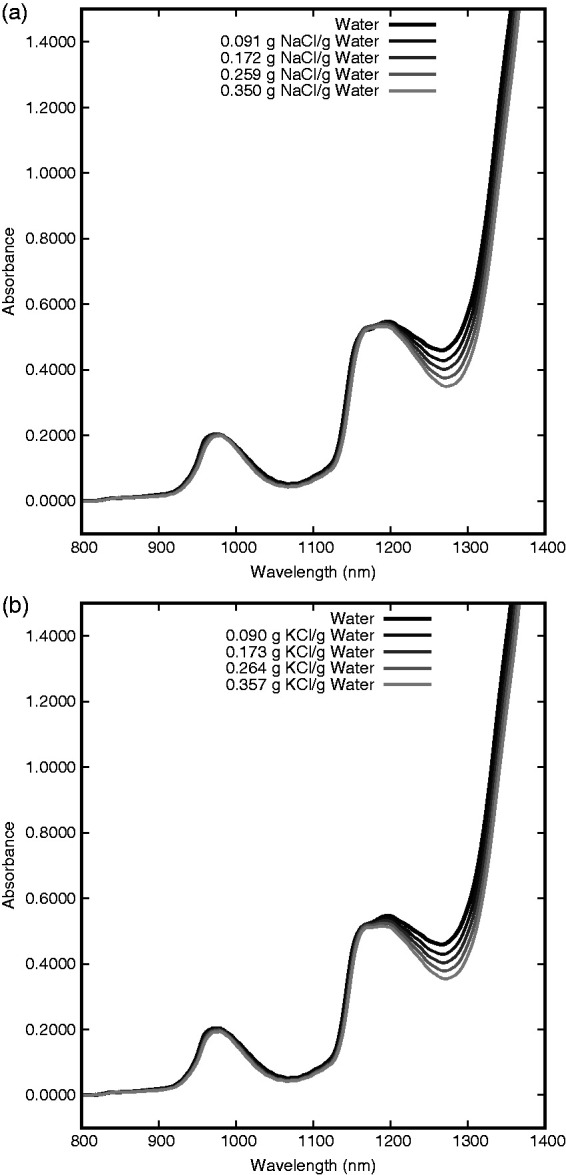
Near infrared spectra at 25℃ of pure water and NaCl aqueous solutions (a) and KCl aqueous solutions (b) at four concentrations.

Two major absorption band centers are seen between 800 and 1400 nm as shown in Figure 1(a) and (b) for NaCl and KCl, respectively. The spectral changes induced by the two salt species are similar, but do exhibit differences in this region both in spectral shape and correlation between concentration and absorbance change.

**Figure 2.**
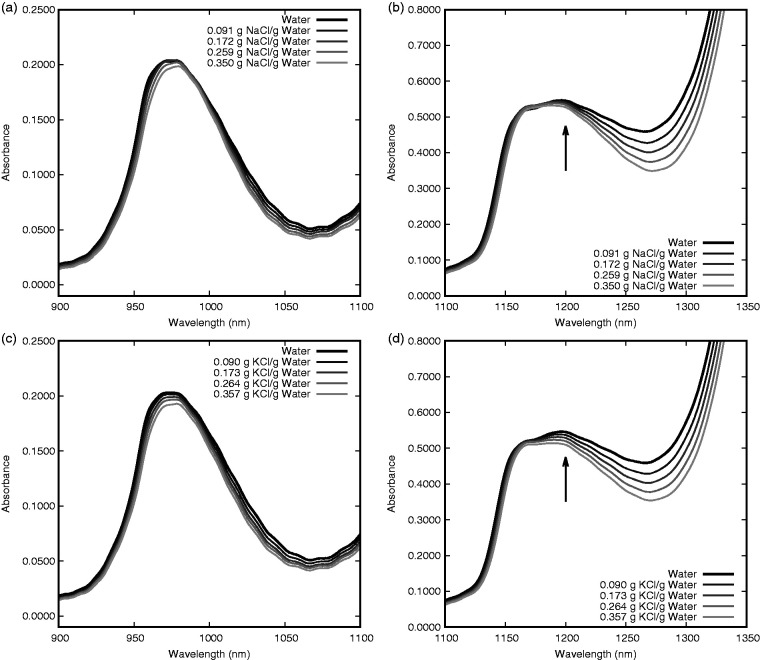
Absorbance band of NaCl near 970 nm (a), NaCl near 1200 nm (b), KCl near 970 nm (c) and KCl near 1200 nm (d). All spectra are of solutions at 25℃. The arrows in (b) and (d) indicate the wavelength where the absorption decrease is more prominent with KCl than with NaCl.

The absorption bands situated near 970 nm are shown in Figure 2(a) and (c) for NaCl and KCl, respectively. This band is dependent on concentration for both NaCl and KCl and corresponds to the second overtone of the symmetric and asymmetric OH-stretching bands.^17^ As the salt concentration increases, the absorbance of this composite band decreases and shifts to a longer wavelength. This effect is similar to the effect observed during a decrease in the temperature of pure water. This could indicate a decrease in the number of water molecules with weak hydrogen bonds according to Abe’s analysis of the absorption decomposition at this band.^7^

The second absorbance band observed in this region occurs near 1200 nm as shown in Figure 2(b) and (d) for NaCl and KCl, respectively. This band is also dependent on both NaCl and KCl concentration, but unlike the band at 970 nm this band corresponds to a combination of the first overtone of the OH-stretching bands and the OH-bending band.^17^ Similar to the 970 nm band, absorbance decreases with an increase in salt concentration. This absorbance decrease is present for both NaCl and KCl between 1150 and 1350 nm, but the decrease is more prominent with KCl at 1200 nm. The shift that is seen with the 970 nm band is less prominent at 1200 nm, possibly due to the combination of all three water vibrations. The decreased absorbance from both the 970 nm peak and the 1200 nm peak cause the region between the two peaks to decrease in absorbance as well.

**Figure 3.**
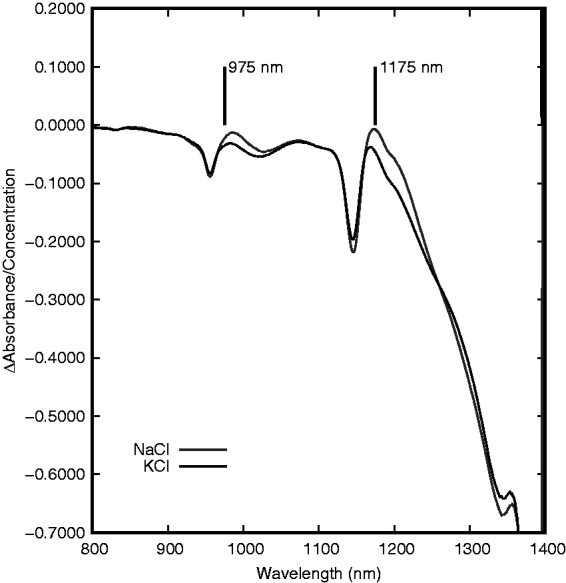
Wavelength-specific linear regression of NaCl and KCl concentrations in aqueous solutions with NIR absorbance at 25℃.

At the 970-nm band, the spectra of these different salt species appear to have more similarities on the shorter wavelength side of the band peak and more differences on the longer wavelength side. The same can be noted for the band near 1200 nm as KCl causes a greater decrease in absorbance on the longer wavelength side of the peak than does NaCl. These differences are not easily visualized when comparing the spectra that include water as the water absorption bands are more substantial than the changes induced by the salt. In order to provide a better comparison of these differences, a linear regression was performed at each wavelength for NaCl and KCl concentrations. The wavelength-dependent slopes (Δ absorbance/concentration in g/g_water_) are displayed for both NaCl and KCl in Figure 3. In this figure, there are a number of features that are more evident than in the regular solution spectra. Congruent with the findings from the previous figures, NaCl and KCl appear to have the greatest absorbance differences on the longer wavelength side of each absorbance peak. These differences are noted near 975 and 1175 nm with KCl producing a greater decrease in absorbance (for a similar concentration). Another notable deviation between the two salt species occurs near 1350 nm. This feature appears on the edge of the high-absorbance harmonic centered near 1470 nm so the full shape and explanation are difficult to ascertain. Many of the differences between the NaCl and KCl in this region could potentially be used for discrimination between the two species as will be discussed further in the section of this work regarding the development of spectral correlations.

## Developing correlations to spectral measurements

Differentiation between single-salt solutions of NaCl and KCl involved estimating the concentration of the sample using a species-independent wavelength then using this concentration to determine expected absorbance values at wavelengths that depend on both salt species and concentration. This method exploits differences in the spectra and uses a closest-fit classification approach to determine the species. It is also analytically very simple and can possibly be done using only two wavelengths. As noted previously, there were a number of wavelength ranges that could be used for differentiating between NaCl and KCl in solution. To determine the best wavelength ranges to use, correlations between absorbance and concentration were calculated through the range of the spectrum. This was done by obtaining the coefficient of determination (*R*^2^) of the linear regression of absorbance and concentration at each wavelength individually.

Between 1230 and 1360 nm, there was very strong correlation between the concentration and absorbance for both species individually and in a combined dataset. High correlation with both species combined indicates that a region is sensitive to concentration, but insensitive to the particular species present. Such a region could therefore be effective for determining overall salt concentration independent of speciation. In comparison, *R*^2^ values in the regions between 1160 and 1230 nm show strong absorbance correlations for the individual salt species, but poor correlation for the aggregated data. This indicates that the region between 1160 and 1230 nm could be useful for species differentiation or an overall salinity check with salt discrimination. There are also many regions in which absorbance is poorly correlated with concentrations of both NaCl and KCl. These poor correlations allowed for quick rejection of many wavelength regions when considering the optimum ranges for wavelength selection.

Wavelengths were sorted by *R*^2^ to determine the best wavelength regions for modelling. For the aggregated data model, the g/g_water_ concentration provided better correlation with the absorbance than the molarity over the majority of the wavelength range. In addition to determining the best correlation, it was important to consider absorbance sensitivity in determining the best wavelength range to be used. The three wavelengths with the highest correlation were 1353, 1256, and 1135 nm. These three wavelengths along with 953 nm, another high correlation wavelength, were compared to evaluate their relative sensitivity.

**Figure 4.**
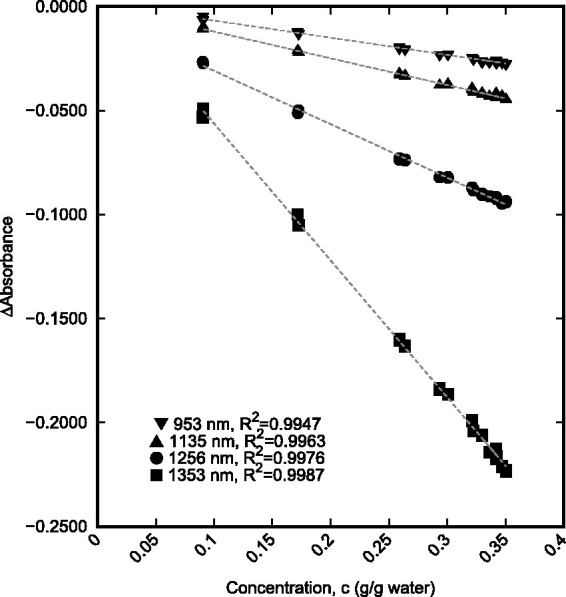
Linear relationship between concentration and absorbance for combined single-salt solution data at four wavelengths, 953 nm (▾), 1135 nm (▴), 1256 nm (•), and 1353 nm (▪).

Figure 4 shows the linear relationship between the g/g_water_ concentration and absorbance that is present at the chosen wavelengths for the aggregate set of NaCl and KCl solution data. Using the g/g_water_ concentration of the solutions at these wavelengths allows for a generic relationship between absorbance and concentration of either NaCl or KCl. The results indicate that (among these wavelengths) the absorbance is most sensitive to changes in salt concentration at 1353 nm as this correlation has the highest slope magnitude.

**Figure 5.**
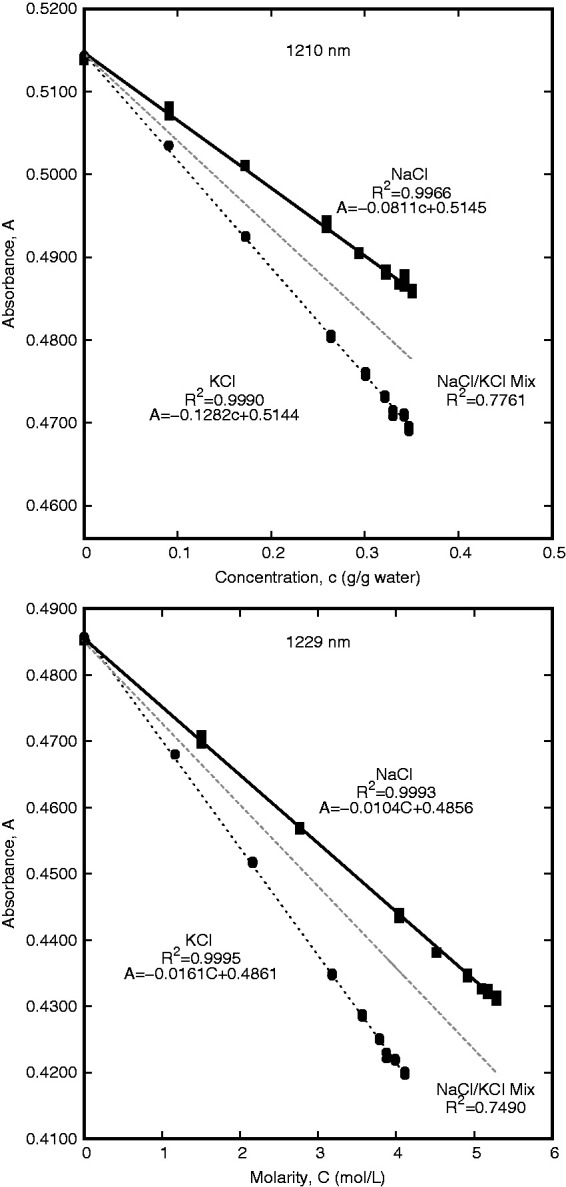
Correlations between concentration and absorbance for pure samples and overall salinity. The wavelength with high correlation between gċgwater^−1^ concentration and absorption was 1210 nm and the wavelength with high correlation between molarity and absorption was 1229 nm.

Regions which have good correlation between absorption and concentration for individual salts but have poor correlation for both species were examined for species differentiation. As with the overall salinity, the correlation values for NaCl and KCl were separately sorted to determine which wavelengths provided the best relationship between concentration and absorbance for each salt. Focus was placed on the 1160–1230 nm range to find wavelengths with good correlation to each species individually, but relatively poor correlation when applied to the aggregate data. The wavelength that was found to have a high g/g_water_ concentration correlation for both NaCl and KCl was 1210 nm. This wavelength provided a coefficient of determination of 0.9990 for KCl and 0.9966 for NaCl, but only 0.7761 for the combined data. Using the molarity correlations, 1229 nm was found to have a coefficient of determination value of 0.9995 for KCl and 0.9993 for NaCl. Due to the differences in maximum molar concentration between NaCl and KCl, the intercept for line of best fit of the aggregate single-salt data had an offset at 1229 nm. The zero-concentration intercept was forced to the absorbance value for deionized water to correct this offset and a coefficient of determination of 0.7490 was found for both datasets. These results are summarized in Figure 5.

Comparing the eight wavelengths that were considered for salt-independent salinity determination, the best correlation value is seen for 1353 nm with the g/g_water_ concentration. This wavelength also has the highest sensitivity and the absorbance at this point is within the reasonable measurement capability of most spectrophotometers using a 10-mm path length (between 1 and 2 absorbance units). These three criteria make 1353 nm a good option for overall salinity determination. For discriminating between NaCl and KCl, the absorbance at 1210 nm and the g/g_water_ concentration was used. A separate calculation for determining molarity used 1229 nm.

### Correlation calibration

Three spectral measurements were taken for each concentration of KCl and NaCl. Two of these measurements were used to develop the equations used for species and concentration determination and the third was used to test the correlations. Thirty-two salt-containing samples and three pure water samples were used to calibrate equations (1) through (5). Equation (1) uses the absorbance measurement at 1353 nm to determine the overall salinity concentration. Using the calculated concentration, equations (2) and (3) describe the expected absorbance values for NaCl or KCl. These expected absorbance values were then compared to the measured absorbance at 1210 nm and the salt species is identified based on the closest fit. Once the salt species was identified, equations (4) or (5) was used to determine the molarity of the NaCl or KCl solution, respectively.

(1) c^XCl=-1.5622×A1353+2.2400

(2) A^1210,NaCl=-0.0811×c^XCl+0.5145

(3) A^1210,KCl=-0.1282×c^XCl+0.5144

(4) C^NaCl=-96.525×A1229+46.869

(5) C^KCl=-62.150×A1229+30.209

where *c_XCl_* is the salt-water mass ratio (*X* is either K or Na), *A* is absorbance at the subscript wavelength specified, and *C* is molar concentration. The hat operator denotes empirically modeled, as opposed to measured values.

### Correlation validation

The correlations developed in equations (1) through (5) were tested using 16 salt-containing samples and two pure water samples. Identifying single-salt solutions required a simple classification of salt species followed by a calculation to determine the molar concentration of the resulting species, if desired. For the range of samples tested (between 25% of the maximum salinity and complete saturation) the correlation correctly identified the salt species in all cases. If the spectral correlation found that the salinity of any given solution was less than 3% of the maximum salinity, it would assume the sample to be pure water. This specification limited the sensitivity of the correlation to samples which had a salinity higher than 3% maximum saturation at 20℃ or 0.16 molar for NaCl and 0.13 molar for KCl.

The results are summarized in Table 3. Error values were calculated on a concentration full-range basis (excluding the water sample). The best average error between actual and estimated salinity was 0.9% for molarity at 1229 nm. The errors with the g/g_water_ concentration were also low having an average of 1.7% and 1.4% for 1210 nm and 1353 nm (overall determination), respectively. The maximum errors in each category were 3.5%, 6.4%, and 3.1% for 1229, 1210, and 1353 nm, respectively. A leave-one-out cross-validation was also performed over the entire calibration and validation datasets totaling 53 samples. This cross-validation resulted in average errors of 1.0%, 2.0%, and 1.5% for 1229, 1210, and 1353 nm, respectively.

**Table 3.** Preliminary results for solutions at 25℃.

Sample type	Sample molarity (mol/L)	Estimated molarity (1229 nm) (mol/L)	% Error	Sample concentration (g/100 gwater)	Estimated concentration (1210 nm) (g/100 gwater)	% Error	Estimated concentration (1353 nm)^a^ (g/100 gwater)	% Error
Water	0.000	0.035	1.0	0.000	0.930	3.6	0.772	3.0
Water	0.000	−0.024	0.7	0.000	0.201	0.8	0.618	2.4
NaCl	1.504 ± 0.001	1.479^b^	0.7	9.077 ± 0.001	8.614^c^	1.8	8.526	2.1
NaCl	2.765 ± 0.002	2.771^b^	0.2	17.188 ± 0.001	16.389^c^	3.1	16.376	3.1
NaCl	4.039 ± 0.004	4.082^b^	1.1	25.901 ± 0.002	25.806^c^	0.4	25.780	0.5
NaCl	4.520 ± 0.005	4.582^b^	1.6	29.349 ± 0.002	29.336^c^	0.0	29.373	0.1
NaCl	4.916 ± 0.005	4.881^b^	0.9	32.257 ± 0.002	31.869^c^	1.5	32.544	1.1
NaCl	5.095 ± 0.005	5.101^b^	0.1	33.624 ± 0.002	34.102^c^	1.8	34.157	2.1
NaCl	5.176 ± 0.005	5.111^b^	1.7	34.217 ± 0.002	32.561^c^	6.4	34.546	1.3
NaCl	5.286 ± 0.005	5.278^b^	0.2	35.048 ± 0.002	35.440^c^	1.5	35.515	1.8
KCl	1.169 ± 0.001	1.128^d^	1.4	9.031 ± 0.001	8.607^e^	1.7	9.048	0.1
KCl	2.164 ± 0.002	2.147^d^	0.6	17.251 ± 0.001	17.170^e^	0.3	17.104	0.6
KCl	3.181 ± 0.003	3.198^d^	0.6	26.385 ± 0.002	26.648^e^	1.0	26.192	0.8
KCl	3.570 ± 0.004	3.578^d^	0.3	30.065 ± 0.002	30.045^e^	0.1	29.766	1.2
KCl	3.786 ± 0.004	3.778^d^	0.3	32.137 ± 0.002	31.923^e^	0.8	31.778	1.4
KCl	3.875 ± 0.004	3.978^d^	3.5	32.997 ± 0.002	33.993^e^	3.9	32.728	1.0
KCl	3.989 ± 0.004	3.999^d^	0.4	34.202 ± 0.002	34.011^e^	0.7	33.764	1.7
KCl	4.115 ± 0.004	4.131^d^	0.5	35.710 ± 0.002	35.015^e^	1.2	35.097	1.5
Average % error			0.9%			1.7%		1.4%
RMSE		0.0387			0.0599		0.0431	

a From equation (1).

b From equation (4).

c From equation (2).

d From equation (5).

e From equation (3).

**Figure 6.**
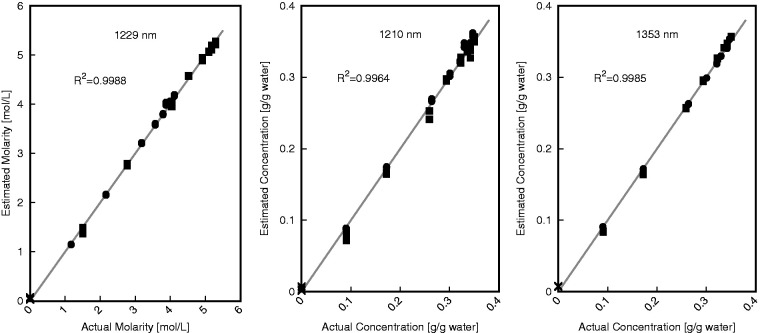
Scatter plot summary of actual and estimated concentrations for aggregate single-salt data at 1229 nm (high correlation between molarity and absorption using equations (4) and (5) for NaCl and KCl, respectively), 1210 nm (high correlation between gċgwater^−1^ concentration and absorption using equations (2) and (3) for NaCl and KCl, respectively), and 1353 nm (high correlation between gċgwater^−1^ concentration and absorption, using equation (1)). Pure water (×) and solutions containing KCl (•) and NaCl (▪) are shown.

Scatter plots comparing actual and estimated values were generated for each wavelength of these three wavelengths is shown in Figure 6. To demonstrate the predictive abilities over the entire set of data collected all 53 samples are shown in this figure. All three scatter plots shown in Figure 6 indicate that these correlations are capable of predicting the salinity of NaCl and KCl solutions. Consistent with the average error values found for each wavelength, 1229 nm produces the best estimation with a coefficient of determination of 0.9988 for the entire set of collected data. Both 1353 and 1210 nm have good coefficients of determination as well with values of 0.9985 and 0.9964, respectively for the entire set of collected data. The plot at 1210 nm indicates that samples with higher concentrations are more difficult to estimate accurately using this wavelength as there is greater deviation at this end. This reduction in accuracy does not appear to be present to the same degree when using 1229 or 1353 nm for concentration estimation. Although using 1210 nm to estimate concentration does not produce results that are as accurate as when using 1229 or 1353 nm, this wavelength still provides very important information regarding speciation of the sample.

## Conclusions

The use of spectroscopy for analyzing NaCl and KCl in solutions has been studied previously in the low concentration range, but extension to saturation and differentiation between these two species in solution has not been investigated extensively. The research presented in this work developed correlations for differentiating between NaCl and KCl in single-salt solutions and determining concentration of NaCl and KCl using spectral measurements in the near infrared range. Differentiating between single-salt solutions resulted in 100% correct species identification that was sensitive to 0.16 molar for NaCl and 0.13 mole per liter for KCl. These correlations were able to determine the concentration of single-salt solutions with an average error of 0.9%. This method allows for species and concentration determination using a few select wavelengths and does not require sophisticated analytical techniques. Future work will include differentiating between NaCl and KCl in dual-salt samples and accounting for temperature variation.
